# Cadmium-Induced Oxidative Damage and the Expression and Function of Mitochondrial Thioredoxin in *Phascolosoma esculenta*

**DOI:** 10.3390/ijms252413283

**Published:** 2024-12-11

**Authors:** Shenwei Gu, Xuebin Zheng, Xinming Gao, Yang Liu, Yiner Chen, Junquan Zhu

**Affiliations:** 1 Key Laboratory of Aquacultural Biotechnology, Ministry of Education, Ningbo University, Ningbo 315211, China; 2 Key Laboratory of Marine Biotechnology of Zhejiang Province, College of Marine Sciences, Ningbo University, Ningbo 315211, China

**Keywords:** *Phascolosoma esculenta*, cadmium, mitochondrial thioredoxin, oxidative stress, apoptosis

## Abstract

*Phascolosoma esculenta* is a unique aquatic invertebrate native to China, whose habitat is highly susceptible to environmental pollution, making it an ideal model for studying aquatic toxicology. Mitochondrial thioredoxin (Trx2), a key component of the Trx system, plays an essential role in scavenging reactive oxygen species (ROS), regulating mitochondrial membrane potential, and preventing ROS-induced oxidative stress and apoptosis. This study investigated the toxicity of cadmium (Cd) on *P. esculenta* and the role of *P. esculenta* Trx2 (*Pe*Trx2) in Cd detoxification. The results showed that Cd stress altered the activities of T-SOD and CAT, as well as the contents of GSH and MDA in the intestine. After 96 h of exposure, histological damages such as vacuolization, cell necrosis, and mitophagy were observed. Suggesting that Cd stress caused oxidative damage in *P. esculenta.* Furthermore, with the prolongation of stress time, the expression level of intestinal *Pe*Trx2 mRNA initially increased and then decreased. The recombinant *Pe*Trx2 (r*Pe*Trx2) protein displayed dose-dependent redox activity and antioxidant capacity and enhanced Cd tolerance of *Escherichia coli*. After RNA interference (RNAi) with *Pe*Trx2, significant changes in the expression of apoptosis-related genes (*Caspase*-3, *Bax*, *Bcl*-2, and *Bcl*-*XL*) were observed. Proving that *Pe*Trx2 rapidly responded to Cd stress and played a vital role in mitigating Cd-induced oxidative stress and apoptosis. Our study demonstrated that *Pe*Trx2 is a key factor for *P. esculenta* to endure the toxicity of Cd, providing foundational data for further exploration of the molecular mechanisms underlying heavy metal resistance in *P. esculenta*.

## 1. Introduction

Cadmium (Cd), a non-essential metal, is widely distributed in aquatic environments [1]. It has gained considerable attention owing to its high toxicity, long biological half-life, and tendency to bioaccumulate in organisms [2]. Studies have revealed that the molecular mechanisms of Cd toxicity are complex and diverse, involving oxidative stress [3,4], apoptosis [5,6], mitochondrial dysfunction [7,8], protein misfolding [9], DNA damage [10], and inflammation [11].

As a redox-inert metal, Cd induces oxidative stress via several indirect pathways. For instance, it binds to the sulfhydryl group of GSH, reducing enzyme activity and weakening the cellular antioxidant capacity [12]. It also disrupts mitochondrial membrane potential, interferes with the electron transport chain, and hinders ATP production, leading to elevated ROS levels in the mitochondria [13]. Furthermore, Cd affects the metabolism of Fe^2+^, promoting the Fenton reaction and resulting in the formation of hydroxyl radicals that exacerbate oxidative damage in organisms [14]. It has been reported that *Litopenaeus vannamei* exhibited increased levels of antioxidant enzyme activities and mucosal damage under Cd stress [15]. After 19 days of Cd exposure, *Micropterus salmoides* showed decreased villus height and density in the intestine [16]. Moreover, Cd stress significantly increased MDA levels in the intestine of *Pelteobagrus fulvidraco*, leading to histological damages, such as increased goblet cells, vacuolization, and thickened lamina propria [17].

To minimize the adverse effects of oxidative damage, organisms have developed several antioxidant systems, with the thioredoxin (Trx) system being one of the most important [18]. Trx is the key component of the Trx system that is found in both prokaryotes and eukaryotes [19]. During redox reactions, the two cysteine residues in its conserved catalytic active site, “CGPC”, interact with disulfide bonds in target proteins, facilitating the reduction of the target proteins and undergoing oxidation itself [20]. Based on its cellular localization, Trx is classified into cytoplasmic Trx (Trx1) and mitochondrial Trx (Trx2). To date, studies on Trx2 have been conducted in aquatic organisms, such as *Haliotis discus discus* [21], *Ruditapes philippinarum* [22], *Mytilus galloprovincialis* [23], *Euphausia superba* [24], and *Hippocampus abdominalis* [25], with a focus on its role in immunity. However, its role in responding to heavy metal stress requires further investigation.

*Phascolosoma esculenta* is a unique aquatic invertebrate native to China, primarily distributed across the intertidal beaches of the southeastern coast [26]. As a zoobenthic species, its habitat is highly susceptible to environmental pollution, which makes it an ideal model for studying aquatic toxicology [27].

In this study, enzyme activity assays, paraffin sectioning, and transmission electron microscopy were used to investigate the changes in intestinal oxidative stress indicators and tissue structure of *P. esculenta* under Cd stress. Additionally, rapid amplification of cDNA ends PCR (RACE-PCR), real-time quantitative PCR (RT-qPCR), and prokaryotic expression were used to examine the response of *P. esculenta* Trx2 (*Pe*Trx2) to Cd stress. Our findings demonstrated that *Pe*Trx2 is a key factor for *P. esculenta* to endure the toxicity of Cd and accumulated basic data for studying the molecular mechanisms of *P. esculenta* to heavy metals.

## 2. Results

### 2.1. Changes of Intestinal Oxidative Stress Indicators of P. esculenta Under Cd Stress

**Figure 1:**
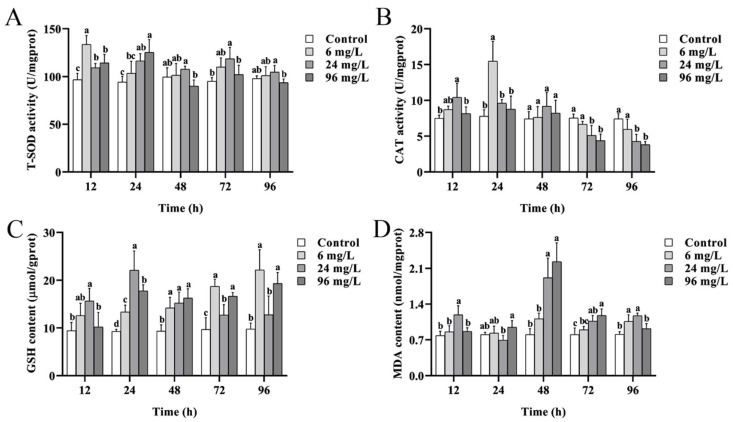
Changes of oxidative stress indicators in the intestine of *P. esculenta* under Cd stress. (**A**) T-SOD activity. (**B**) CAT activity. (**C**) GSH content. (**D**) MDA content. Data were shown as mean ± SD (*n* = 3). Different letters indicate significant differences among the groups (*p* < 0.05).

T-SOD activity was induced under Cd stress. After 12 h and 24 h of Cd stress, it was significantly higher than that of the control group (*p* < 0.05) (Figure 1A). CAT activity was increased after 12 h and 24 h of Cd stress, followed by a decrease as the stress duration lengthened. In the 24 and 96 mg/L groups, it was significantly lower than that of the control group at 72 h and 96 h (*p* < 0.05) (Figure 1B). GSH content was elevated under Cd stress and was higher than that of the control group throughout the experiment (Figure 1C). MDA content was gradually increased under Cd stress, peaking at 48 h, and then decreased (Figure 1D).

### 2.2. Changes of Intestinal Structure of P. esculenta Under Cd Stress

**Figure 2:**
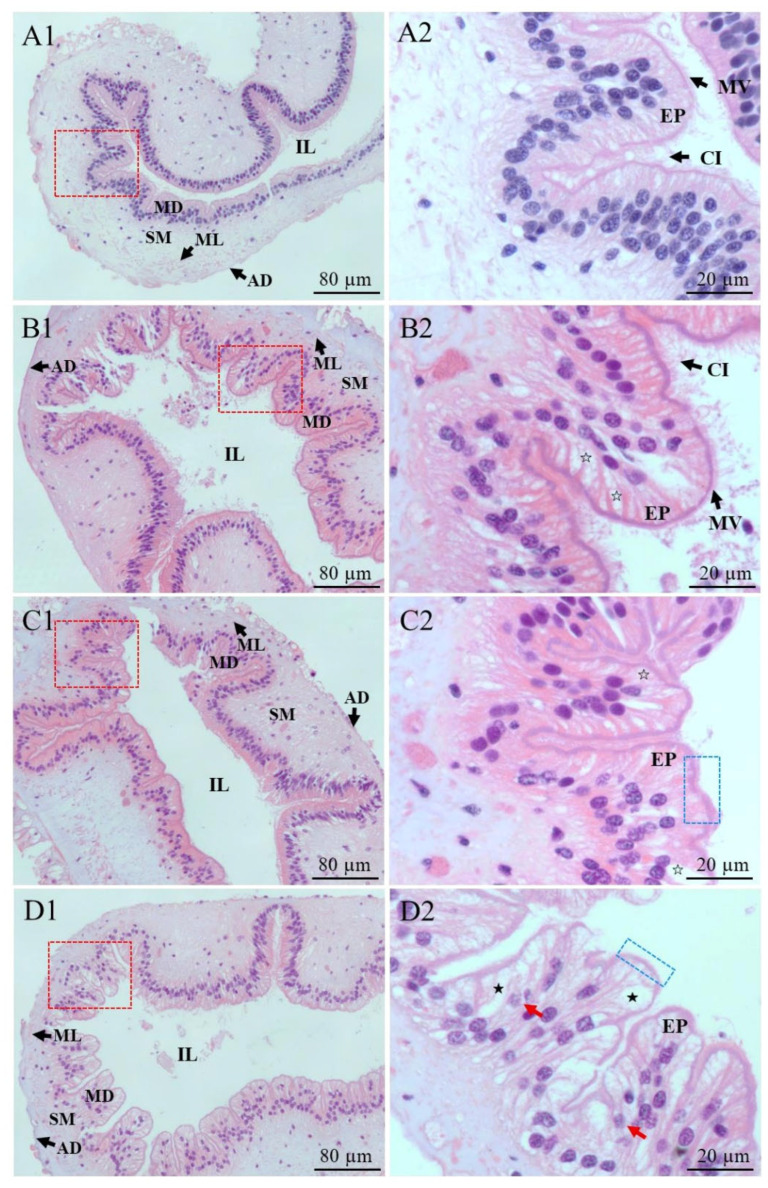
Microstructural changes in the intestine of *P. esculenta* after 96 h of Cd stress. (**A1**,**A2**) Cross-sectional structure of the intestine in the control group. The morphology of epithelial cells is normal, and the structures of cilia and microvilli are clearly visible. (**B1**,**B2**) Cross-sectional structure of the intestine in the 6 mg/L group. The epithelial cells are vacuolated (white pentagram), but the structures of cilia and microvilli remain clear. (**C1**,**C2**) Cross-sectional structure of the intestine in the 24 mg/L group. The epithelial cells are vacuolated (white pentagram), with blurred microvilli and disappeared cilia (blue box). (**D1**,**D2**) Cross-sectional structure of the intestine in the 96 mg/L group. The epithelium cells are severely vacuolated (black pentagram), with necrotic cells (red arrows), blurred microvilli, and disappeared cilia (blue box). Red box shows A2, B2, C2, and D2, respectively. MD: mucosal fold; SM: submucosa; ML: muscle layer; AD: adventitia; IL: intestinal lumen; EP: epithelium cell; CI: cilia; MV: microvilli.

Histological changes in the intestine of *P. esculenta* under Cd stress are shown in Figure 2. In the control group, the intestinal structure was intact, with tightly arranged, single-layer columnar epithelial cells and densely distributed microvilli and cilia. However, in the Cd-treated groups, varying degrees of vacuolization were observed in the epithelial cells. As Cd concentrations increased, the damage became more severe in the 24 and 96 mg/L groups, including blurred microvilli and loss of cilia. Furthermore, necrotic cells were observed in the 96 mg/L group. Histopathological scores were shown in Table 1.

**Figure 3:**
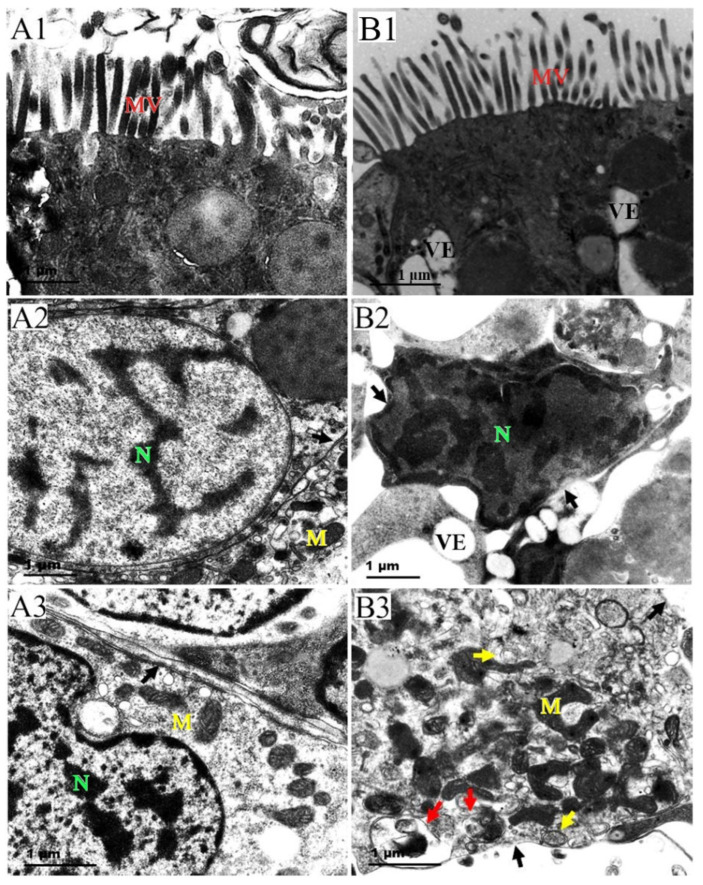
Ultrastructural changes in the intestine of *P. esculenta* after 96 h of Cd stress. (**A1**) Control group, 15,000×, the microvilli of the intestinal epithelial cells are densely arranged. (**A2**) Control group, 20,000×, the nucleus is regular, the nuclear membrane structure is clear, and the cell membrane is intact (black arrow). (**A3**) Control group, 25,000×, the mitochondrial cristae is clear, the morphological structure is normal, and the cell membrane is intact (black arrow). (**B1**) 96 mg/L group, 12,000×, the microvilli on the free surface of intestinal epithelial cells was still intact, while numerous vacuoles appeared in the cells. (**B2**) 96 mg/L group, 20,000×, the nuclear membrane structure was damaged (black arrow), with condensed chromatin accumulating near the inner side of the nuclear membrane. (**B3**) 96 mg/L group, 20,000×, the cristae are blurred, a large number of autophagosomes (red arrow) and autophagy-like vesicles (yellow arrow) appeared in cells, and the nuclear membrane structure is damaged (black arrow). MV: microvilli, N: nucleus, M: mitochondria, VE: vacuole.

Ultrastructural changes in the intestine of *P. esculenta* under Cd stress are shown in Figure 3. In the control group, the microvilli of the epithelial cells were intact and densely arranged. The nucleus exhibited a regular shape with a complete nuclear membrane, evenly distributed euchromatin, and scattered heterochromatin clumps. Moreover, the mitochondrial cristae were clearly visible. In the 96 mg/L group, the microvilli structure remained intact, whereas vacuoles appeared in the epithelial cells. The nuclear membrane structure showed signs of damage, with condensed chromatin accumulating near its inner surface. Mitochondrial cristae were significantly blurred, and autophagosomes and autophagy-like vesicles were present.

### 2.3. Characterization of PeTrx2

**Figure 4:**
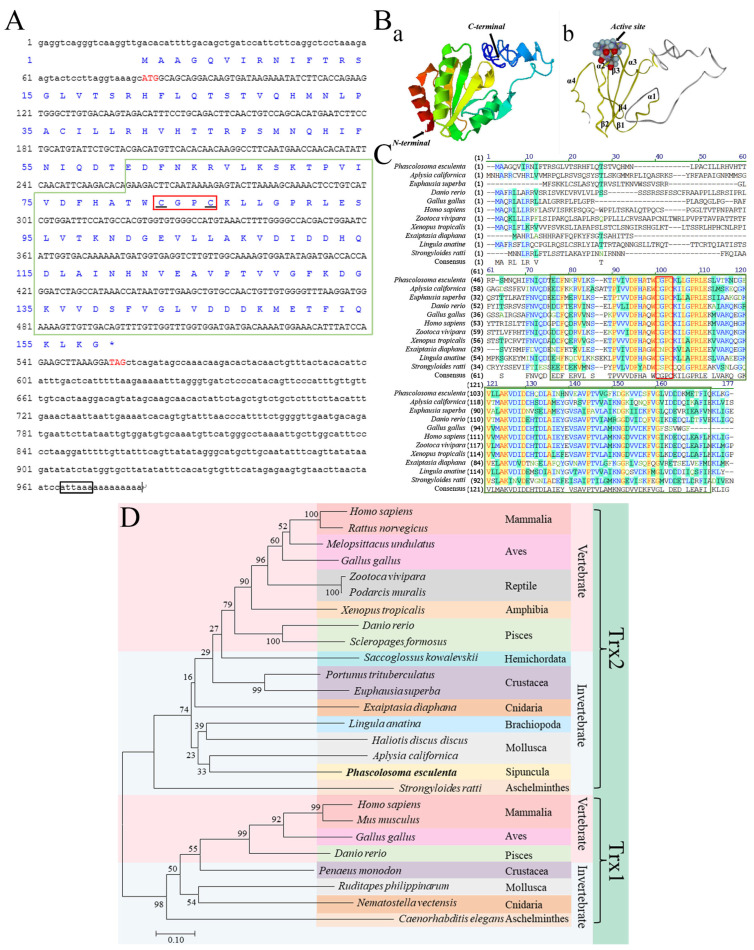
Characterization of *Pe*Trx2. (**A**) Full-length cDNA sequence and deduced amino acid sequence of *Pe*Trx2. The start codon and stop codon are marked with red fonts, the polyadenylate tail signal is marked with a black box, the redox-active site “CGPC” is marked with a red box, the black underlines represent conservative cysteines, and the Trx domain is marked with a green box. (**B**) The 3D structure of *Pe*Trx2. (**a**) The N- and C-terminal of *Pe*Trx2. (**b**) The yellow part shows the Trx domain, which has an active site. (**C**) Multiple sequence alignment of *Pe*Trx2 and its homologs. The red box indicates the conserved redox active site, and the green box indicates the Trx domain. (**D**) Phylogenetic tree analysis of *Pe*Trx2. *P. esculenta* is shown in bold font, and *Pe*Trx2 belongs to the invertebrate branch.

The cDNA of *Pe*Trx2 was 979 bp in length, consisting of a 79 bp 5′-untranslated region (5′-UTR), a 423 bp 3′-UTR, and a 477 bp open reading frame (ORF) encoding 158 amino acids (Figure 4A). The predicted isoelectric point and molecular weight were 7.09 and 17.69 kDa, respectively. No signal peptide was found, and *Pe*Trx2 was predicted to be located in the mitochondria. The 3D structure of *Pe*Trx2 contains a typical Trx domain, comprising four α-helixes and four β-sheets, and arranged in the order of α1β1α2β2α3β3β4α4 (Figure 4B). The redox active site “CGPC” is located at the N-terminus of the second α-helix. Sequence alignment showed that *Pe*Trx2 shared low identity with Trx2 homologs from *Homo sapiens*, *Gallus gallus*, *Zootoca vivipara*, *Xenopus tropicalis*, *Danio rerio*, *E. superba*, *Lingula anatine*, *Aplysia californica*, *Strongyloides*, and *Exaiptasia diaphana*. However, their Trx domain shared higher identity (Table 2, Figure 4C). Phylogenetic analysis showed that Trx2 and Trx1 are divided into two branches, with *Pe*Trx2 belonging to the Trx2 branch (Figure 4D).

### 2.4. Tissue-Specific Expression of PeTrx2 and Its Expression Changes Under Cd Stress

**Figure 5:**
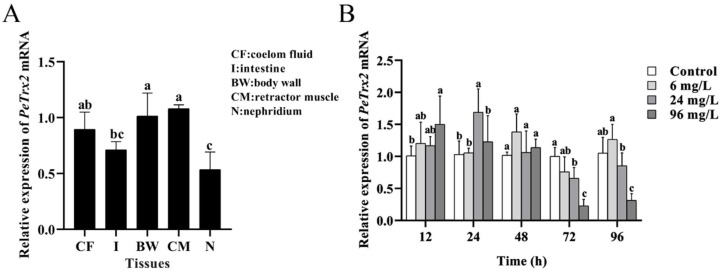
Tissue-specific expression of *Pe*Trx2 mRNA and its expression changes under Cd stress. (**A**) Expression of *Pe*Trx2 mRNA in different tissues. CF: coelom fluid, I: intestine, BW: body wall, CM: retractor muscle, N: nephridium. (**B**) The relative expression level of *Pe*Trx2 mRNA in the intestine of *P. esculenta* following Cd stress. Data were shown as mean ± SD (*n* = 3). Different letters indicate significant differences among the tissues or groups (*p* < 0.05).

*Pe*Trx2 mRNA was expressed in all tissues, showing as retractor muscle > body wall > coelom fluid > intestine > nephridium (Figure 5A). Under Cd stress, the expression of intestinal *Pe*Trx2 mRNA first increased and then decreased as the prolongation of stress duration, peaking at 12 h (96 mg/L group), 24 h (24 mg/L group), and 48 h (6 mg/L group), respectively. After 72 h and 96 h of stress, the expression levels declined as Cd concentrations increased. In the 24 mg/L group, the *Pe*Trx2 expression level was significantly higher than that of the control group at 24 h and was significantly lower than that of the control group at 72 h and 96 h (*p* < 0.05). Similarly, in the 96 mg/L group, it was significantly higher than the control group at 12 h and was significantly lower than the control group at 72 h and 96 h (*p* < 0.05) (Figure 5B).

### 2.5. Expression and Purification of rPeTrx2

**Figure 6:**
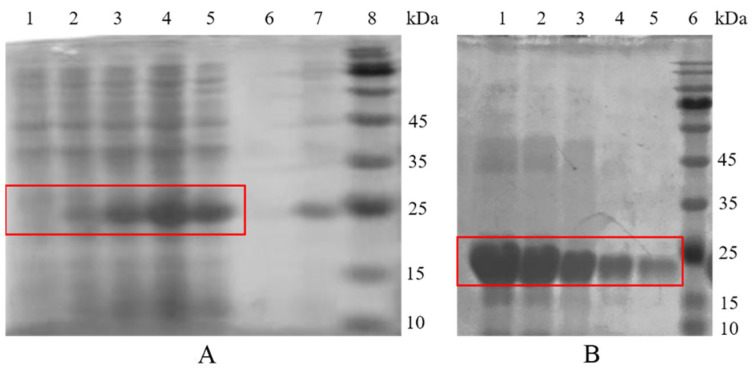
Expression and purification of r*Pe*Trx2. (**A**) Induction effect of r*Pe*Trx2 over time. Line 1: the cell lysate of r*Pe*Trx2 without induction; Line 2: the cell lysate of r*Pe*Trx2 induced for 1 h; Line 3: the cell lysate of r*Pe*Trx2 induced for 3 h; Line 4: the cell lysate of r*Pe*Trx2 induced for 5 h; Line 5: the cell lysate of r*Pe*Trx2 induced for 7 h; Line 6: the supernatant of the cell lysate; Line 7: the precipitation of the cell lysate; Line 8: marker. (**B**) Purification of r*Pe*Trx2. Line 1–5: purified proteins with different imidazole elution ladders; Line 6: marker. The red boxes show the target bands.

The recombinant pET28a-*Pe*Trx2 plasmid was transfected into Transetta (DE3) and expressed under the induction of 1 mM IPTG. SDS-PAGE analysis showed that r*Pe*Trx2 was mainly present in inclusion bodies (Figure 6A). After purification, a single band corresponding to a molecular weight of 15–25 kDa was obtained, which was consistent with the results predicted by the Expasy ProtParam tool (Figure 6B).

### 2.6. In Vitro Antioxidant Activity of rPeTrx2

**Figure 7:**
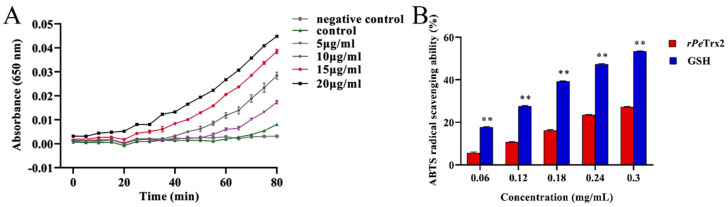
The antioxidant activity of r*Pe*Trx2. (**A**) Insulin disulfide bond reducing activity of r*Pe*Trx2. The absorbance (650 nm) of the reaction mixture was monitored following the addition of DTT. The reaction system without r*Pe*Trx2 was set as the control, and the reaction system without DTT and r*Pe*Trx2 was set as the negative control. (**B**) ABTS radical scavenging activity of r*Pe*Trx2. Different concentrations of GSH were set as the positive control. Data were shown as mean ± SD (*n* = 3). ** represents *p* < 0.01.

As shown in Figure 7A, the Ab_650_ value in the experimental group was positively correlated with the concentration of r*Pe*Trx2 and the duration of the reaction, indicating that r*Pe*Trx2 exhibited dose- and time-dependent redox activity. In contrast, owing to the lack of r*Pe*Trx2, slight changes were observed in the control group, and no changes were detected in the negative control group.

As shown in Figure 7B, the ABTS radical scavenging rates of r*Pe*Trx2 and GSH were positively correlated with their concentrations. At a concentration of 0.3 mg/mL, r*Pe*Trx2 exhibited a scavenging rate of 27.13%, and GSH demonstrated a rate of 53.31%. The half maximal inhibitory concentration (IC_50_) for r*Pe*Trx2 and GSH was determined to be 0.68 mg/mL and 0.27 mg/mL, respectively.

### 2.7. Cd Tolerance of the E. coli Cells Transferred pET28a-PeTrx2

**Figure 8:**
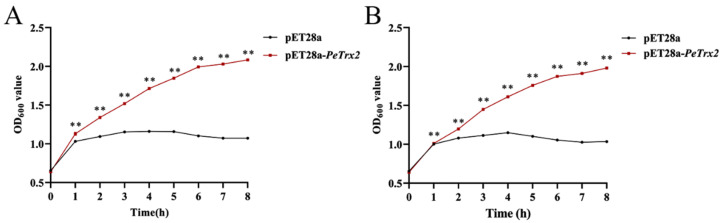
Effects of Cd stress on the growth of pET28a and pET28a-*Pe*Trx2. (**A**) 0.3 mM CdCl_2_ group; (**B**) 0.6 mM CdCl_2_ group. Data were shown as mean ± SD (*n* = 3). ** represents *p* < 0.01.

The OD_600_ values of *E. coli* cells transformed with pET28a and pET28a-*Pe*Trx2 were almost the same at 0 h. Under 0.3 mM and 0.6 mM CdCl_2_ stress, the OD_600_ values of pET28a-*Pe*Trx2 increased consistently over time, with higher OD_600_ values observed in the 0.3 mM group compared to the 0.6 mM group. In contrast, the OD_600_ values of pET28a initially increased and then decreased, showing inhibition at 5 h for the 0.3 mM group and at 4 h for the 0.6 mM group (Figure 8).

### 2.8. The Anti-Apoptosis Ability of PeTrx2

**Figure 9:**
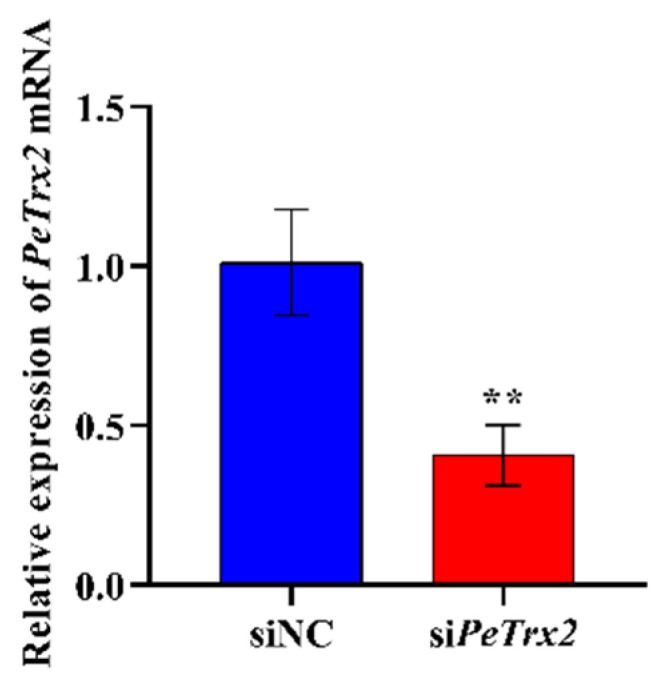
Changes in the relative expression of *Pe*Trx2 mRNA. *GAPDH* was used as an inference. Data were shown as mean ± SD (*n* = 3). ** represents *p* < 0.01.

**Figure 10:**
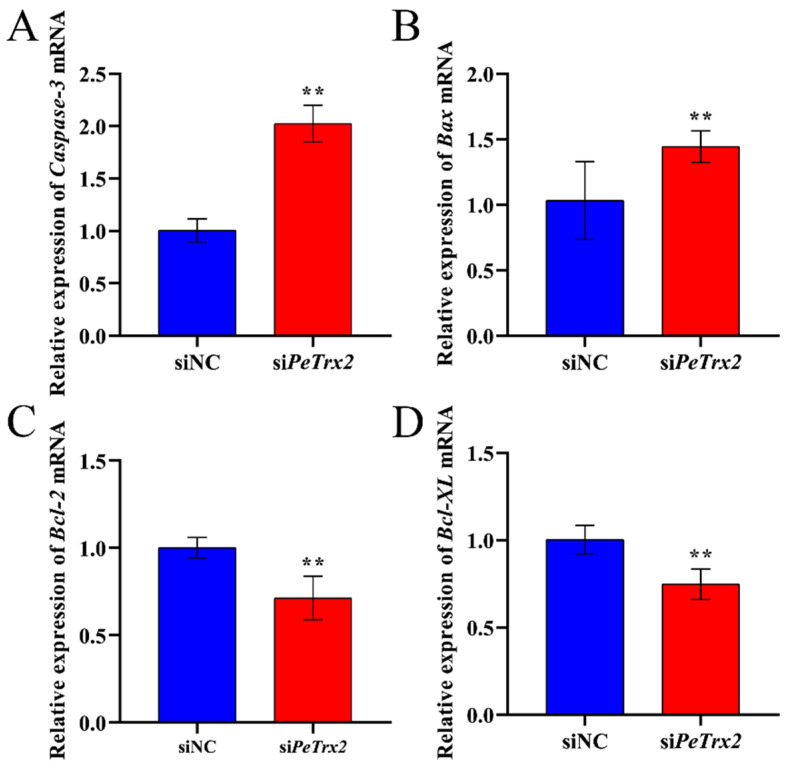
Changes in the relative expression levels of apoptosis-related genes. (**A**) *Caspase*-3. (**B**) *Bax*. (**C**) *Bcl*-2. (**D**) *Bcl-XL*. *GAPDH* was used as an inference. Data were shown as mean ± SD (*n* = 3). ** represents *p* < 0.01.

Compared to the siNC group, the expression level of intestinal *Pe*Trx2 mRNA in the si*Pe*Trx2 group was significantly decreased by 60% (*p* < 0.01) (Figure 9). Meanwhile, the expression levels of *Caspase*-3 and *Bax* mRNA were significantly increased to 202% and 140% of the control group, respectively (*p* < 0.01) (Figure 10A,B). In contrast, the expression levels of *Bcl*-2 and *Bcl-XL* mRNA were significantly decreased to 71% and 75% of the control group, respectively (*p* < 0.01) (Figure 10C,D).

## 3. Discussion

### 3.1. Cd Stress Induced Oxidative Damage in the Intestine of P. esculenta

As a peroxide inducer, Cd triggers oxidative stress by promoting the production of excess ROS via various indirect pathways [14]. Oxidative stress is often accompanied by the activation of antioxidant systems and oxidative damage to cells [28]. SOD, CAT, and GSH are the major antioxidants in the body that maintain redox homeostasis by detoxifying excess ROS. SOD serves as the first line of defense, converting the superoxide anion (O^2−^) into H_2_O_2_ and O_2_ [29], and CAT catalyzes the decomposition of H_2_O_2_ into H_2_O and O_2_. Yuan et al. [30] found that SOD activity in *Perinereis aibuhitensis* initially increased and then decreased under 17.22 mg/L of Cd stress. Lei et al. [31] reported that CAT activity in the heart of *Sinopotamon yangtsekiense* first increased and then decreased over time. In the present study, similar changes in SOD and CAT activities were observed in the intestines of *P. esculenta*, indicating that SOD and CAT are involved in ROS removal during the early stages of Cd stress. However, excessive ROS may inhibit the production of SOD and CAT during prolonged stress [32]. Additionally, Cd was reported to replace Fe^2+^ in the active center of CAT or induce ROS production, which can explain the decrease in CAT activity [33]. GSH is a crucial non-enzymatic antioxidant that directly removes ROS or serves as a substrate for glutathione peroxidase (GSH-Px) to detoxify H_2_O_2_ [34]. It also binds to Cd, forming Cd-GSH complexes, and reducing the toxicity of Cd [14]. Studies have shown that GSH content in *Micropterus salmoides* [16] and *Oreochromis niloticus* [35] significantly increased under Cd stress. In this study, the intestinal GSH content of *P. esculenta* in the treatment groups was induced under Cd study, indicating that GSH plays a vital role in Cd stress resistance. MDA, the product of lipid peroxidation, reflects the degree of oxidative damage [36]. Zhang et al. [37] found that MDA content in *Procambarus clarkia* significantly increased after 48 h and 72 h of Cd stress. Similarly, in this study, the MDA content in the intestine of *P. esculenta* increased after 48 h, which may be the result of peroxidative damage that affects the production of antioxidant enzymes. Based on these findings, we conclude that the antioxidant system was activated to counteract Cd-induced oxidative stress. However, as Cd concentration and stress duration increase, excessive ROS may cause oxidative damage, affecting the production of antioxidant enzymes and resulting in lipid peroxidation.

The accumulation of lipid peroxides may cause structural damage to organisms. Tanhan et al. [38] and Gao et al. [39] found that *Babylonia areolate* and *Daphnia magna* exhibited vacuolization, loss of microvilli, and shortened cilia under Cd stress. Similar results were observed in our study. The movement of microvilli and cilia facilitates the transport of food and residues [40]; their damage may affect the nutrient absorption and waste metabolism in *P. esculenta*. Additionally, studies have shown that excessive ROS can cause oxidative damage to the nucleus and mitochondria [41] and impair the function of Na^+^/K^+^-ATPase, resulting in cellular edema. This accounts for the observed damage, such as concentrated chromatin and vacuolization (Figure 2) [42]. Notably, autophagy was also observed in this study, as autophagic lysosomes encapsulated damaged mitochondria and formed autophagosomes with double-layered membranes (Figure 3), which is the main characteristic of autophagy. After encapsulating the damaged organelles, the double-layered autophagosomes fuse with lysosomes, forming a single-layer autophagosome that removes the damaged organelles [43]. The increase of autophagic lysosomes after Cd stress may be a protective mechanism for the body to cope with Cd stress. However, the appearance of necrotic cells may result from the excessive self-digestion of intracellular components [44]. These findings suggest that Cd stress poses a threat to *P. esculenta*.

### 3.2. Expression and Functional Analysis of PeTrx2

#### 3.2.1. Characterization and Expression Analysis of *Pe*Trx2

Trx is a low-molecular-weight protein with redox activity, widely present in both prokaryotes and eukaryotes [19]. Based on its cellular localization, Trx is classified into cytoplasmic Trx (Trx1) and mitochondrial Trx (Trx2). In vivo, Trx participates in various metabolic processes through the Trx system, which is one of the most critical antioxidant systems in addition to SOD, CAT, and GSH-Px. In this study, the *Trx2* gene of *P. esculenta* (*Pe*Trx2) was cloned and identified. The ORF is 477 bp in length, encoding 158 amino acids. A conserved Trx domain was identified at the C-terminus, and typical Trx-like folding (βαβα and ββα) was found by 3D structure prediction [45], which is characterized by four α-helixes wrapped around four β-sheets and arranged in the order of α1β1α2β2α3β3β4α4. The N-terminus of the second α-helix contains a conserved redox active site, “CGPC”, where the reversible exchange of thiol and disulfide bonds between the Cys residues enables Trx to perform its redox regulation functions [19,46]. Sequence alignment showed that the Trx domain of *Pe*Trx2 is evolutionarily conserved, sharing 45.3% to 64.9% similarity with homologous proteins. Additionally, phylogenetic analysis clustered *Pe*Trx2 in the Trx2 branch. Based on these findings, we identified *Pe*Trx2 as a new member of the Trx superfamily.

Mitochondria are the key target organelles of Cd toxicity. Excessive ROS disrupt the electron transport chain, leading to mitochondrial oxidative damage and triggering mitochondria-dependent apoptosis [47,48]. Trx2, an antioxidant located in the mitochondria, plays a crucial role in scavenging ROS, regulating mitochondrial membrane potential, and preventing ROS-induced apoptosis [49,50]. Studies have shown that *Trx2* mRNA is widely expressed in mammals, with particularly high expression levels in organs with high-energy metabolism, such as the heart, muscles, and kidneys [51,52]. In addition, *Trx2* mRNA has been detected in aquatic animals, including *R. philippinarum* [22], *M. galloprovincialis* [23], and *Sebastes schlegelii* [53], with varying expression levels across species and tissues. In this study, *Pe*Trx2 mRNA was examined in the retractor muscle, body wall, coelom fluid, intestine, and nephridium of *P. esculenta*, showing a small variation in fold differences across tissues, indicating that *Pe*Trx2 may play a critical role in all tissues of *P. esculenta*. Under Cd stress, the expression level of intestinal *Pe*Trx2 mRNA initially increased and then decreased, following a similar pattern as the changes in enzyme activities and the expression level of *Pe*Trx1 [54], indicating that *Pe*Trx2 participates in antioxidant activities together with other antioxidants.

#### 3.2.2. Functional Analysis of *Pe*Trx2

Trx2 is a member of the Trx superfamily, playing vital roles in anti-oxidative stress and regulating mitochondria-dependent apoptosis. Li et al. [24] purified recombinant *E. superba* Trx2 protein and found that it exhibited strong redox activity and antioxidant capacity. De Zoysa et al. [21] constructed a recombinant *Haliotis discus hainai* Trx2 protein that catalyzes the reduction of insulin disulfide bonds through DTT-mediated reactions. Additionally, Nadarajapillai et al. [25] explored the functions of recombinant *H. Abdominalis* Trx2 protein, which has insulin-reducing and DPPH-free radical scavenging activities. Furthermore, after transfection with *HaTrx2*, the survival rate of the FHM cells under H_2_O_2_ stress was significantly increased. In this study, the r*Pe*Trx2 protein displayed dose-dependent redox activity and antioxidant capacity and enhanced the Cd tolerance of *E. coli*, indicating that *Pe*Trx2 plays a critical role in the antioxidant defense system of *P. esculenta*.

Apoptosis is a programmed cell death process regulated by apoptotic genes [55]. Recently, the role of Trx in regulating apoptosis has gained significant attention. Tanaka et al. [50] found that Trx2 deficiency led to ROS accumulation in DT40 cells, which triggered the release of cytochrome C from the mitochondria and activated Caspase-3 and Caspase-9, resulting in apoptosis. Pérez et al. [56] demonstrated that Trx2-deficient mice (Trx2^+/−^) produced less mitochondrial ATP than wild-type mice, along with increased ROS and 8-OHdG levels, leading to an increase in cytochrome C, activation of Caspase-3 and Caspase-9, and induction of cell apoptosis. Liu et al. [57] reported that overexpression of Trx2 in INS-1 cells reduced the production of mitochondrial ROS induced by acetaldehyde and inhibited the activity of ASK1 and p38 MAPK, thereby reducing mitochondrial damage and preventing mitochondrial-dependent apoptosis. In this study, RNAi with *Pe*Trx2 and Cd stress resulted in a significant increase in the expression of *Caspase*-3 and *Bax* mRNA, which promoted apoptosis in the intestine of *P. esculenta*. In contrast, the expression levels of *Bcl*-2 and *Bcl-XL* mRNA, which are anti-apoptotic genes, were significantly decreased. These results suggest that *Pe*Trx2 may resist Cd-induced oxidative stress and apoptosis by scavenging ROS and inhibiting mitochondria-dependent apoptosis, and it plays an important role in the resistance of *P. esculenta* to Cd stress.

## 4. Materials and Methods

### 4.1. Animals

*P. esculenta* individuals used in this study were collected from the coastal tidal zone of Xizhou, Ningbo, Zhejiang, China, with an average weight of 3.9 ± 0.7 g. They were kept indoors and temporarily cultured in filtered seawater at a temperature of 22 ± 0.5 °C, a salinity of 28‰, and a pH of 7.9 for 24 h.

### 4.2. Treatments

The median lethal concentration (LC_50_) of Cd for *P. esculenta* over 96 h was 192 mg/L [50]. Based on this value, four Cd stress concentrations were set for this study, including 0 mg/L (control), 6 mg/L (1/32 96 h LC_50_), 24 mg/L (1/8 96 h LC_50_), and 96 mg/L (1/2 96 h LC_50_). Each group had three replicate tanks, with 50 *P. esculenta* individuals placed in each tank. CdCl_2_·2.5H_2_O (Sinopharm, Shanghai, China) was used to adjust the concentration of Cd in seawater. The seawater was refreshed daily, and the Cd concentration was consistently maintained.

### 4.3. Sampling

Three individuals were randomly selected from the control group, and their coelom fluid, intestine, constrictor muscle, nephridium, and body wall were sampled for tissue-specific expression analysis. After 12, 24, 48, 72, and 96 h of Cd stress, three intestines from each group were collected for enzyme activity assays and RT-qPCR analysis. After 96 h of Cd stress, the superior segment of the ascending intestine from three individuals was sampled, with a portion fixed in Bouin’s solution (Phygene, Fuzhou, China) for histological analysis and the remaining fixed in 2.5% glutaraldehyde (Solarbio, Beijing, China) for ultrastructural observation.

### 4.4. Detection of the Intestinal Oxidative Stress Indicators of P. esculenta Under Cd Stress

The intestine was mixed with chilled saline and homogenized in an ice bath to extract total protein. The mixture was then centrifuged at 12,000 rpm at 4 °C for 8 min, and the supernatant was collected. Protein concentration was assessed using a BCA protein assay kit (CWBIO, Taizhou, China), and SOD and CAT activities, as well as GSH and MDA contents, were measured using commercially available kits (Solarbio, Beijing, China). The operation is carried out according to the instructions.

### 4.5. Observation of the Intestinal Structure of P. esculenta Under Cd Stress

After 24 h of fixation, the superior segment of the ascending intestine was removed from Bouin’s solution. It was then dehydrated using a gradient of alcohol (70%–80%–90%–95%I–95%II–100%I–100%II), cleared with xylene (50%–100%I–100%II), and embedded in paraffin. After solidification, the paraffin blocks were sliced into 7-μm-thick sections, stained with hematoxylin and eosin (H&E), and observed under a microscope (Nikon, Tokyo, Japan). The criteria for judging the degree of damage were modified based on the previous study: no damage (-), damage ≤ 5% (+), 5% < damage ≤ 10% (++), and damage > 10% (+++) [16].

After 2 h of fixation in 2.5% glutaraldehyde and 1% osmium tetroxide, the superior segment of the ascending intestine was taken off and dehydrated using a gradient of alcohol and acetone (70%–85%–100%). Samples were then infiltrated with Epon-812 epoxy resin, sectioned using an ultramicrotome (Leica, Wetzlar, Germany), stained with uranyl acetate and lead citrate, and observed under a transmission electron microscope (Hitachi, Tokyo, Japan).

### 4.6. Characterization and Expression Analysis of PeTrx2

#### 4.6.1. RNA Extraction and cDNA Synthesis

RNA was extracted from the tissues using the RNA-Solv Reagent (Omega, Cambridge, MA, USA). It was then reversed to cDNA using the PrimeScript^®^ RT Kit and SMARTer RACE 5′/3′ reagent (Takara, Dalian, China) for gene cloning and quantitative analysis.

#### 4.6.2. Full-Length cDNA Cloning of *Pe*Trx2

The ORF of *Pe*Trx2 was obtained from transcriptome data. Based on which, primers named *Pe*Trx2-F and *Pe*Trx2-R were designed using Primer Premier 5.0 for verification, and primers used for 5′ and 3′ RACE were designed for full-length cloning (Table S1). PCR amplification, DNA purification, and sequencing were conducted following the methods outlined in our previous study [50].

#### 4.6.3. Sequence Analysis of *Pe*Trx2

ORFfinder (http://www.ncbi.nlm.nih.gov/orffinder/, accessed on 7 July 2021) was used to predict the ORF of *Pe*Trx2, and the sequences were subsequently translated into amino acids using Bioxm 2.6 (Nanjing, China). Expasy ProtParam (http://web.expasy.org/protparam/, accessed 10 August 2021) was used to predict molecular weight and isoelectric point. SignalP-5.0 (http://www.cbs.dtu.dk/services/SignalP/, accessed on 10 August 2021) was used to predict potential signal peptides. CD-search (http://www.ncbi.nlm.nih.gov/Structure/cdd/wrpsb.cgi, accessed on 10 August 2021) was used to identify conserved domains. WoLF PSORT (https://wolfpsort.hgc.jp/, accessed on 10 August 2021) was used to predict the subcellular localization. I-TASSER (https://zhanglab.ccmb.med.umich.edu/I-TASSER/, accessed on 10 August 2021) was used to predict 3D structure. Vector NTI 11.5 (Invitrogen, Waltham, MA, USA) and Mega 7.0 (Informar Technologies, Los Angeles, CA, USA) were used for multiple sequence alignment and phylogenetic tree analysis. GenBank accession numbers of the homologous proteins are listed in Table S2.

#### 4.6.4. Tissue-Specific Expression of *Pe*Trx2 and Its Expression Characteristics Under Cd Stress

Tissue-specific expression of *Pe*Trx2 and its expression changes under Cd stress were detected by RT-qPCR, with GAPDH acting as an internal reference. The reaction system consisted of 7 μL ddH_2_O, 1 μL *Pe*Trx2-F, 1 μL *Pe*Trx2-R, 1 μL cDNA (diluted 30 times), and 10 μL 2×RealStar Green Fast Mixture. The reaction procedures were 95 °C for 5 min and 40 cycles of (95 °C for 15 s, 59 °C for 15 s, 72 °C for 15 s, and 72 °C for 1 s). Results were analyzed by the 2^−ΔΔCT^ method. The primer sequences are listed in Table S1.

### 4.7. Functional Analysis of PeTrx2

#### 4.7.1. Prokaryotic Expression of *Pe*Trx2

The ORF of *Pe*Trx2 was amplified using a forward primer containing *BamH*I (r*Pe*Trx2-F) and a reverse primer containing *Xho*I (r*Pe*Trx2-R). The reaction system consisted of 7 μL ddH_2_O, 1 μL *Pe*Trx2-F, 1 μL *Pe*Trx2-R, 1 μL cDNA, and 10 μL 2 × Super Pfx MasterMix. The reaction procedures were 98 °C for 1 min, 35 cycles of (98 °C for 10 s, 52 °C for 20 s, and 72 °C for 30 s), and 72 °C for 10 min. The PCR product and pET28a vector were digested with *BamH*I and *Xho*I (Thermo Fisher Scientific, Waltham, MA, USA), respectively, and ligated using T4 ligase (Takara, Dalian, China) to construct the recombinant pET28a-*Pe*Trx2 plasmid. It was then transformed into Transetta (DE3) cells (TransGen Biotech, Beijing, China) and spread on the solid LB medium (containing 100 μg/mL kanamycin (Kana)). After overnight culture at 37 °C, positive clones were selected and sequenced. The *E. coli* cells transformed with pET28a-*Pe*Trx2 were cultured in the liquid LB medium (containing 100 μg/mL Kana) at 37 °C and 180 rpm until the OD_600_ reached 0.6. After which, isopropyl-β-d-1-thiogalactopyranoside (IPTG) at a final concentration of 1 mM was added to induce the expression of the recombinant protein. After 0, 1, 3, 5, and 7 h of induction, 1 mL of bacterial solution was centrifuged at 12,000 rpm to collect the cells containing the target protein. It was then mixed with 5×SDS-PAGE sample loading buffer and denatured for 10 min. Finally, the induction effect was analyzed by SDS-PAGE electrophoresis.

#### 4.7.2. Purification and Renaturation of *Pe*Trx2

After 8 h of induction, the cells were collected and purified using a His-tag protein purification kit (inclusion body protein) according to the instructions (CWBIO, Taizhou, China). The purified protein was then dialyzed in 50 mM PBS containing decreasing concentrations of urea (6, 4, 3, 2, 1, and 0 M) for refolding, with the addition of 10% glycerin, 0.02 mM GSSG, and 2 mm GSH to enhance the refolding efficiency (except for the 50 mM containing 0 M urea). Finally, the protein was concentrated by poly(ethylene glycol) 20,000, and the concentration was determined using the Bradford protein assay kit (Solarbio, Beijing, China).

#### 4.7.3. In Vitro Antioxidant Activity of r*Pe*Trx2

To assess the insulin disulfide reduction activity of r*Pe*Trx2, a 600 μL mixture was prepared, consisting of 402 μL of PBS (50 mM), 100 μL of insulin (2 mg/mL), 12 μL of EDTA (10 mM), 6 μL of DTT (100 mM), and 80 μL of r*Pe*Trx2 at varying concentrations. In the control group, an equal volume of 50 mM PBS was used to replace the r*Pe*Trx2, while in the negative control group, both r*Pe*Trx2 and DTT were replaced with an equal volume of 50 mM PBS. The mixtures were incubated at 25 °C for 80 min, with absorbance at 650 nm (Ab_650_) measured every 5 min.

The ABTS radical scavenging ability of r*Pe*Trx2 at different concentrations (0.1, 0.2, 0.3, 0.4, and 0.5 mg/mL) was evaluated using a total antioxidant capacity assay kit (Solarbio, Beijing, China) following the ABTS method. GSH and PBS at the same concentrations served as positive and negative controls, respectively, and an equal volume of distilled water acted as the blank control. Each sample was measured three times at Ab_414_.

#### 4.7.4. Cd Tolerance of the E. coli Cells Transferred pET28a-*Pe*Trx2

The pET28a and pET28a-*Pe*Trx2 bacteria strains were cultured in liquid LB medium containing 100 μg/mL Kana until the OD_600_ reached 0.6. At that point, 1 mM IPTG was added to induce protein expression, followed by the addition of 0.3 mM and 0.6 mM CdCl_2_. The bacteria were cultured at 37 °C for 8 h, with OD_600_ measured every hour.

#### 4.7.5. The Anti-Apoptosis Ability of *Pe*Trx2

RNA interference (RNAi) was performed according to the method described by Gu et al. [58]. Lipo6000^TM^ (Beyotime, Shanghai, China), siRNA (GenePharma, Shanghai, China), and 10 mM PBS were mixed in a volume ratio of 17:17:6. The mixture was injected at a dose of 1 µg/g. After 24 h of RNAi treatment and 24 h of Cd stress, the intestines were collected. The interference efficiency and expression changes of apoptosis-related genes (*Caspase*-3, *Bax*, *Bcl*-2, and *Bcl-XL*) were then assessed by RT-qPCR. The interference primers are listed in Table S1.

### 4.8. Statistical Analysis

Statistical analysis was conducted by Excel 2016 and SPSS 26.0. One-way ANOVA and Duncan’s test were used to assess the significance of oxidative stress indicators and *Pe*Trx2 expression changes across 4 groups, with 3 replicates in each group. Additionally, the independent samples *t*-test was applied to evaluate the significance of other datasets. *p* < 0.05 was considered statistically significant.

## 5. Conclusions

This study investigated the acute toxicity of Cd stress on the intestines of *P. esculenta* and the role of *Pe*Trx2 in Cd detoxification. The findings revealed that Cd stress caused changes in enzyme activities and damaged the intestinal structure of *P. esculenta*, resulting in oxidative damage. At the same time, the expression level of intestinal *Pe*Trx2 mRNA initially increased and then decreased, indicating that it was rapidly responsive to Cd stress. Additionally, recombinant *Pe*Trx2 protein demonstrated in vitro antioxidant activity, and *E. coli* transfected *Pe*Trx2 exhibited increased tolerance to Cd. After RNAi with *Pe*Trx2 and Cd stress, changes in the expression level of apoptosis-related genes were observed, proving that *Pe*Trx2 plays a critical role in the antioxidant and anti-apoptosis of *P. esculenta*.

## Figures and Tables

**Table 1:** Histopathological changes in the intestine of *P. esculenta* after 96 h of Cd stress.

Lesion	Degree of Damage			
	Control	6 mg/L	24 mg/L	96 mg/L
Vacuolization	-	+	+	+++
Blurred microvilli	-	-	+++	+++
Cilia loss	-	-	++	+++
Necrosis	-	-	-	+
Autophagy	-	/	/	+

Note: no damage (-); damage ≤ 5% (+); 5% < damage ≤ 10% (++); damage > 10% (+++); not detected (/).

**Table 2:** Identity between *Pe*Trx2 and its homologous proteins.

Specie	Identity of Trx2	Identity of Trx Domain
*Homo sapiens*	45.8%	62.1%
*Gallus gallus*	40.5%	56.8%
*Zootoca vivipara*	45.7%	63.2%
*Xenopus tropicalis*	41.3%	60.0%
*Danio rerio*	41.0%	55.8%
*Euphausia superba*	42.8%	56.8%
*Lingula anatine*	46.7%	64.9%
*Aplysia californica*	40.2%	60.8%
*Strongyloides*	32.1%	45.3%
*Exaiptasia diaphana*	38.6%	53.7%

## Data Availability

The data that have been used are confidential.

## Supplementary Materials

The following supporting information can be downloaded at: https://www.mdpi.com/article/10.3390/ijms252413283/s1.
