# Supplementary material for: Cadmium-Induced Oxidative Damage and the Expression and Function of Mitochondrial Thioredoxin in Phascolosoma esculenta
Source: Int J Mol Sci. 2024 Dec 11;25(24):13283. doi: 10.3390/ijms252413283 (PMC11676412; doi:10.3390/ijms252413283)
Supplement: Supplementary file 1 [file ijms-25-13283-s001.zip › Table S1.pdf]

Table S1. The primers and their sequences

Primer	Sequence(5'-3')	Purpose
PeTrx2-F	CACCAGAAGTGGGCTTGT	PCR
PeTrx2-R	GCCAACAAGACCTCACCAT	PCR
5’*PeTrx2*-R1	TCACCAATGATTCCAGTCGTGGC	5’RACE
5’ *PeTrx2*-R2	GCAGGAAGATTCATGTGCTGGACAGT	5’RACE
3’ *PeTrx2*-F1	GTGGGCTTGTGACAAGTAGACAT	3’RACE
3’ *PeTrx2*-F2	GGATTTCCATGCCACGTGGT	3’RACE
q*PeTrx2*-F	TCCTGCATGTATTCTGCTACG	RT-qPCR
q*PeTrx2*-R	CACCAATGATTCCAGTCGTG	RT-qPCR
GAPDH-F	CCAGAACATCATCCCAGCA	RT-qPCR
GAPDH-R	ACGAACAGGGACACGGAAG	RT-qPCR
r*PeTrx2*-F	CGC**GGATCC**ATGGCAGCAGGACAAGTGAT	Prokaryotic expression
r*PeTrx2*-R	CCG**CTCGAG**TCCTTTAAGCTTCTGGATAAAT	Prokaryotic expression
si*PeTrx2*-sense	GGACAAGUGAUAAGAAAUATT	RNA inference
si*PeTrx2*-antisense	UAUUUCUUAUCACUUGUCCTT	RNA inference
siNC-sense	UUCUCCGAACGUGUCACGUTT	RNA inference
siNC-antisense	ACGUGACACGUUCGGAGAATT	RNA inference
Caspase-3-F	ATCAGCCACCCTCAAACC	RT-qPCR
Caspase-3-R	TGCCCTCCCTCTCACTCT	RT-qPCR
Bax-F	GACGCAGGGCAAATGATA	RT-qPCR
Bax-R	TGTAGGTGGGCAATGGAG	RT-qPCR
Bcl-2-F	TGATGGAGTAAAATGGGGTC	RT-qPCR
Bcl-2-R	TCCTTGCTGTCAGGGTTG	RT-qPCR
Bcl-XL-F	AGACCGCCCAAAACCTAC	RT-qPCR
Bcl-XL-R	TTTCATTTACAGTCCTCCTCAG	RT-qPCR
