# Supplementary material for: Cadmium-Induced Oxidative Damage and the Expression and Function of Mitochondrial Thioredoxin in Phascolosoma esculenta
Source: Int J Mol Sci. 2024 Dec 11;25(24):13283. doi: 10.3390/ijms252413283 (PMC11676412; doi:10.3390/ijms252413283)
Supplement: Supplementary file 1 [file ijms-25-13283-s001.zip › Table S2.pdf]

Table S2. Species and GenBank accession numbers

Specie	Protein	GenBank accession number
Homo sapiens	Trx1	AAF86466.1
Mus musculus	Trx1	NP_035790.1
Gallus gallus	Trx1	NP_990784.1
Danio rerio	Trx1	NP_001002461.1
Penaeus monodon	Trx1	ADV36299.1
Ruditapes philippinarum	Trx1	AET44428.1
Caenorhabditis elegans	Trx1	NP_001021886.1
Nematostella vectensis	Trx1	XP_001638202.1
Homo sapiens	Trx2	AAF86467.1
Rattus norvegicus	Trx2	NP_445783.1
Melopsittacus undulatus	Trx2	AAO72715.1
Gallus gallus	Trx2	NP_001026581.1
Zootoca vivipara	Trx2	XP_034984086.1
Podarcis muralis	Trx2	XP_028602716.1
Xenopus tropicalis	Trx2	NP_001008161.1
Danio rerio	Trx2	NP_991204.1
Scleropages formosus	Trx2	XP_018612453.1
Saccoglossus kowalevskii	Trx2	XP_002733252.1
Portunus trituberculatus	Trx2	AFE88627.1
Euphausia superba	Trx2	AMK05618.1
Lingula anatina	Trx2	XP_013417621.1
Haliotis discus discus	Trx2	ABO26636.1
Aplysia californica	Trx2	XP_005100694.1
Strongyloides ratti	Trx2	XP_024507212.1
Exaiptasia diaphana	Trx2	XP_020900392.1
