# Supplementary figures and images for: Cadmium-Induced Oxidative Damage and the Expression and Function of Mitochondrial Thioredoxin in Phascolosoma esculenta
Source: Int J Mol Sci. 2024 Dec 11;25(24):13283. doi: 10.3390/ijms252413283 (PMC11676412; doi:10.3390/ijms252413283)

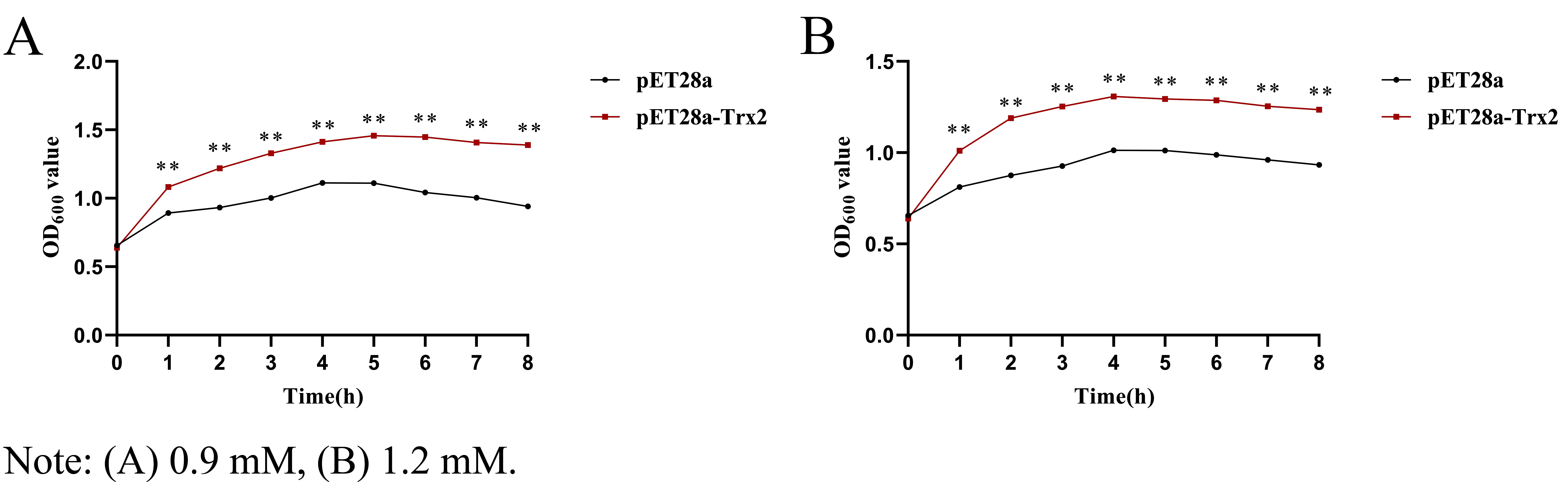

Supplement: Supplementary file 1 [file ijms-25-13283-s001.zip › Figure S1.tif]
